# The Importance of the Expendable: Toxin–Antitoxin Genes in Plasmids and Chromosomes

**DOI:** 10.3389/fmicb.2017.01479

**Published:** 2017-08-04

**Authors:** Ramón Díaz-Orejas, Manuel Espinosa, Chew Chieng Yeo

**Affiliations:** ^1^Centro de Investigaciones Biológicas, Consejo Superior de Investigaciones Científicas (CSIC) Madrid, Spain; ^2^Faculty of Medicine, Biomedical Research Centre, Universiti Sultan Zainal Abidin Kuala Terengganu, Malaysia

**Keywords:** toxin–antitoxin, plasmids, post-segregational killing, genomic islands, chromosome, bacterial virulence, persistence

## Abstract

Toxin–antitoxin (TA) genes were first reported in plasmids and were considered expendable genetic cassettes involved in the stable maintenance of the plasmid replicon by interfering with growth and/or viability of bacteria in which the plasmid was lost. TAs were later found in bacterial chromosomes and also in integrated mobile genetic elements; they were proposed to be involved in the bacterial response to stressful situations. At present, 100s of TAs have been identified and classified in up to six families (I to VI), with those belonging to the type II (constituted by two protein components) being the most studied. Based on well-characterized examples of several type II TAs, we discuss in this review that irrespective of their locations in plasmids or chromosomes, TAs functionally overlap as indicated by: (i) in both locations they can mediate the maintenance of genetic elements to which they are physical linked, and (ii) they can induce persistence or virulence in response to stress situations. Examples of functional confluences in homologous TA systems with different locations are also given. We also consider whether the physiological role of TAs is due to their genetic organization as operons or to their inherent properties, like the short lifespan of the antitoxin component.

## Introduction

Toxin–antitoxin (TA) genes were initially discovered in two conjugative plasmids of *Escherichia coli*, F (Ogura and Hiraga, 1983) and R1 (Gerdes et al., 1986a; Bravo et al., 1988; Tsuchimoto et al., 1988), as cassette of two genes involved in stable maintenance of these plasmids; they were shown to participate in stable plasmid inheritance because they reduced either the viability or the growth of the cells that had lost the plasmid at the time of cell division (Bravo et al., 1988; Tsuchimoto et al., 1988). Killing of plasmid free segregants was termed post-segregational killing (PSK) (Gerdes et al., 1986b) and shown to be due to the decay of the more unstable antitoxin in plasmid-free cells and to the subsequent activation of the toxin in these cells. A role of plasmidic TAs in outcompeting compatible plasmids was later proposed (Cooper and Heinemann, 2000). TAs were also subsequently shown to be encoded by bacterial chromosomes and some of them integrated within mobile genetic elements (MGEs). One of the hypotheses to explain the presence and function of these TAs was that they participate in the response to stressful conditions (Christensen and Gerdes, 2003). At present, 100s of TAs have been identified and classified, depending on the nature and activity of antitoxins, in up to six types (type I to type VI), reviewed in Page and Peti (2016). To these toxin–antitoxin pairs can also be added Type II restriction enzymes and their cognate methylases: similar to conventional TAs, restriction-modification systems can also be encoded in plasmids and enforce their maintenance by promoting PSK of plasmid-free cells (Yarmolinsky, 1995), although it must be emphasized that TA and RM complexes that have been characterized so far do not share an evolutionary origin (Mruk and Kobayashi, 2014). Type II TAs, which are constituted by two protein components, are the best characterized (Chan et al., 2016; Kędzierska and Hayes, 2016). Activation of TAs in response to stress was thought to be a way to either eliminate part of the population in benefit of the rest [i.e., “altruistic” cell death (Engelberg-Kulka and Glaser, 1999)], or to reduce the metabolic load during adverse conditions by slowing or arresting cell growth (Maisonneuve and Gerdes, 2014). Chromosomal TAs have also been associated with several bacterial processes, like biofilm formation, survival during infection of eukaryotic cells, defense against invading bacteriophages and entrance and exit into persistence (Goeders and Van Melderen, 2014; Kędzierska and Hayes, 2016; Lobato-Márquez et al., 2016a). Since persistence is believed to be a major factor contributing to the chronic state of infections and tolerance to antibiotic treatments (Michiels et al., 2016), it was proposed that one of the roles of TAs was to contribute to dormancy, i.e., making the cells metabolically inactive (Pedersen et al., 2002; Christensen-Dalsgaard et al., 2010), a state that would lead to persistence due to triggering of the TAs (Maisonneuve et al., 2011; Maisonneuve and Gerdes, 2014). This attractive hypothesis was later considered as too simplistic (Ramisetty et al., 2016; Van Melderen and Wood, 2017). Since the initial discovery of TA systems as plasmid maintenance systems and their subsequent identification in the chromosome, many examples underline the functional confluence of these systems irrespective of their location. In the following sections we will try do discuss this confluence based on a few well-characterized examples of type II TA systems (see **Table 1** for a summary of the TA systems covered in this review).

**Table 1:** Summary of the location and functions of toxin–antitoxin (TA) systems covered in this review.

TA	Host	Location of TA	Function	Reference
MosAT	*Vibrio cholerae*	Chromosomal; integrative and conjugative element (ICE)	Stability of ICE	Wozniak and Waldor, 2009
SgiTA	*Salmonella enterica* serovar Typhimurium	Chromosomal; genomic island 1	Stability of genomic island	Huguet et al., 2016
RelBE1 ParDE1	*Vibrio vulnificus*	Chromosomal; superintegron	Superintegron stability	Szekeres et al., 2007
ParDE-1 ParDE-2 ParDE-3 RelBE-1 RelBE-2 RelBE-3 RelBE-4 HigBA-1 HigBA-2 Phd-Doc 0318-0319 0332-0333 0422-0423 0477-0478 0481-0482 0486-0487 0488-9489	*Vibrio cholerae*	Chromosome II, superintegron	Stability of superintegron as well as chromosome II	Iqbal et al., 2015
Epsilon-Zeta (𝜀-ζ)	*Streptococcus pyogenes*	Plasmid pSM19035	Plasmid stability	Camacho et al., 2002
PezAT	*Streptococcus pneumoniae*	Chromosomal, pathogenicity island 1	Stability of pathogenicity island, virulence factor	Chan et al., 2014; Iannelli et al., 2014
SezAT	*Streptococcus suis*	Chromosomal; pathogenicity island	Stability of pathogenicity island	Yao et al., 2015
AvrRxo1-Arc1	*Xanthomonas oryzae* pv. *oryzicola*	Unknown	Virulence factor (type III secreted effector)	Triplett et al., 2016
CcdAB_F_	*Escherichia coli*	Plasmid F	Plasmid stability, persistence	Ogura and Hiraga, 1983; Tripathi et al., 2012
CcdAB_O157_	*Escherichia coli* O157:H7	Chromosomal	Persistence	Gupta et al., 2017
VapBC2_ST_ CcdAB_ST_	*Salmonella enterica* serovar Typhimurium	Plasmid pLST	Plasmid stability, virulence Plasmid stability	Lobato-Márquez et al., 2016b

## Plasmid and Chromosomal Toxin–Antitoxins as Mediators of Post-Segregational Killing

Several observations indicate the existence of functional overlaps of TAs placed on plasmids or on chromosomes. A role of the chromosomal TAs in stabilization of integrated MGE or adjacent chromosomal regions has been demonstrated (Wozniak and Waldor, 2009), a role that is similar to their function in maintaining plasmid stability through PSK (Hayes, 2003). A novel TA pair designated *mosAT*, was shown to be responsible for maintaining the integrity of the ∼100 kb SXT integrative and conjugative element (ICE) that confers resistance to multiple antibiotics in clinical isolates of *Vibrio cholerae* (Wozniak and Waldor, 2009). For a large MGE that can integrate, excise, and transfer to other bacteria, the SXT ICE is remarkably stable, with loss of ICE estimated at only 1 in 10^7^ cells (Wozniak and Waldor, 2009). The *mosAT* system has low basal transcriptional levels when SXT is integrated; however its expression is derepressed when SXT is in an extrachromosomal state and vulnerable to loss. Interestingly, a homolog of *mosAT* was located on an octopine-type Ti plasmid of *Agrobacterium tumefaciens* suggesting that this TA system may function to maintain the stability of plasmids as well as ICEs (Wozniak and Waldor, 2009). Another TA system designated *sgiTA* was recently shown to promote the maintenance of a multidrug resistant integrative and mobilizable *Salmonella* Genomic Island 1 (SGI1) in *Salmonella enterica* serovar Typhimurium (Huguet et al., 2016) in a manner similar to *mosAT* for SXT. Intriguingly, SGI1 is only transmissible in the presence of conjugative plasmids of the IncA/C group but paradoxically, SGI1 displayed incompatibility with the IncA/C plasmids. The *sgiTA* locus was shown to play an essential role in SGI1 stability particularly in the concomitant presence of a conjugative IncA/C plasmid when SGI1 is in an extrachromosomal state and is more likely to be lost (Huguet et al., 2016).

Further, elimination of cells that lose a chromosome has been demonstrated in the case of *V. cholerae* (Yuan et al., 2011). Like all vibrios, the *V. cholerae* genome consists of two chromosomes (Heidelberg et al., 2000), and the smaller chromosome II (ChrII, 1.07 Mbp) hosts a large 126 kb superintegron (SI) that gathers 100s of diverse gene cassettes, including antibiotic resistance genes, and contains 17 TA systems (Iqbal et al., 2015). Each of these cassettes is associated with a target recombination sequence (the *attC* site) and in a SI, 100s of gene cassettes with their *attC* sites are arranged in direct orientation, alluding to a likely inherent instability in these SIs. Nevertheless, SIs are remarkably stable, and in an earlier paper, it was elegantly demonstrated that two TA loci from the *Vibrio vulnificus* SI (*relBE1* and *parDE1*) stabilize the SI and prevent large scale deletions from occurring when the SI was devoid of TA loci (Szekeres et al., 2007). Chromosome-specific mechanisms exist to ensure the proper segregation of the two *V. cholerae* chromosomes in daughter cells. In the *V. cholerae* ChrII, the *parAB2* locus was essential for the partitioning of ChrII, and in a *parAB2* deletion mutant, ChrII was mislocalized leading to a complete loss of the entire ChrII in a fraction of the population (Yamaichi et al., 2007). Cells that lost ChrII were non-viable and underwent characteristic cytological changes including cell enlargement, nucleoid condensation and degradation (Yamaichi et al., 2007). It was subsequently shown that the three ParE toxins encoded by their respective *parDE* TA loci in the SI of ChrII were responsible for the PSK of cells that lost ChrII, closely mimicking PSK mediated by plasmid-encoded homologs (Yuan et al., 2011). A recent paper showed that all 17 TA loci in the *V. cholerae* SI were functional, expressed from their own native promoters, and were very specific – i.e., there was no cross-interaction between non-cognate toxins and antitoxins (Iqbal et al., 2015). These advocate for a major role of these 17 TA loci in the stabilization of the *V. cholerae* SI and to prevent the emergence of cells that lack ChrII; in other words, much like their plasmid-encoded homologs, the *V. cholerae* chromosomal TA loci are also agents of PSK (Yuan et al., 2011; Iqbal et al., 2015).

Conversely, it has been shown that plasmid-encoded TAs can contribute to overcome stress, induce persistence, and could increase survival of bacterial cells during infection (Helaine et al., 2014), functions that were initially attributed to chromosomal TAs (Lobato-Márquez et al., 2016a). PSK mediated by TAs following the loss of genetic information associated to a plasmid can be considered as a situation of stress, to which the cells react by toxin activation. Transient activation of the *E. coli* F-plasmid-encoded CcdB toxin enhance the generation of drug-tolerant persister cells, and this process was found to be dependent on Lon protease and RecA (Tripathi et al., 2012). The F-plasmid-encoded *ccdAB*_F_ locus has been well-established as a plasmid maintenance system (Jaffe et al., 1985) and the finding that it plays a role in persistence expands its function as a transmissible persistence factor (Tripathi et al., 2012) (see below).

## PezAt and its Potential Role in Virulence

Two recent articles published in *Frontiers* (Chan and Espinosa, 2016; Lobato-Márquez et al., 2016b) underline the concept that phenotypes associated to plasmid- or to chromosomally encoded TAs do overlap because independent of their location, toxins target similar functions and the TA operons are regulated and induced by similar conditions. The first example is provided by the pneumococcal *pezAT* operon (Khoo et al., 2007; Chan and Espinosa, 2016). The two genes constituting it are placed in the putative mobilizable pathogenicity island 1 (pneumococcal pathogenicity island 1, PPI1) and that is found in nearly half of capsulated (virulent) *Streptococcus pneumoniae* strains (Chan et al., 2012). In some strains there is a second copy of the operon, located on the putative ICE Tn*5253*. A close homolog of the PezAT pair is the Epsilon-Zeta TA, which was discovered in the broad host-range plasmid pSM19035 of *Streptococcus pyogenes* (Camacho et al., 2002). Epsilon-Zeta differs from PezAT as the Epsilon antitoxin does not perform the transcriptional regulation of the operon, but rather by a third component, Omega, which also regulates the transcription of other genes encoded by the pSM19025 plasmid. In the *pezAT* operon, the PezA antitoxin performs the autoregulatory role (Khoo et al., 2007). Epsilon-Zeta plays an essential role in maintaining the stability of plasmid pSM19035 via PSK.

The Zeta/PezT toxins target the cell wall synthesis machinery by phosphorylating the peptidoglycan precursor, UDP-*N*-acetylglucosamine (UNAG) at the 3′-OH group of the *N*-acetylglycosamine moiety. The phosphorylated product, UDP-*N*-acetylglucosamine-3-phosphate (UNAG-3P), accumulates in the cytosol and inhibits MurA, which is the essential enzyme that catalyzes the initial step in peptidoglycan synthesis (Mutschler and Meinhart, 2011; Mutschler et al., 2011). Nevertheless, it was proposed that reduction in the UNAG levels is just one of several responses that is triggered by Zeta/PezT expression in response to stress (Tabone et al., 2014). The *pezAT* operon may play a role in stabilizing the MGE within the pneumococcal host (Chan et al., 2014; Iannelli et al., 2014). Further, a close homolog of *pezA*T was discovered in *Streptococcus suis* (designated *sezAT*), which was shown to be important for the stable inheritance of the Pathogenicity Island 1 (SsPI-1) (Yao et al., 2015). Pneumococcal strains that carry *pezAT* exhibit increased virulence; further, deletion of the operon led to pneumococcal cells exhibiting increased resistance to β-lactam antibiotics and to increased ability to take up homologous DNA by enhancing genetic competence (Chan and Espinosa, 2016). How PezT functions to increase pneumococcal virulence is currently unknown but it was postulated that activation of PezT during environmental stresses or the course of infection would result in inhibition of cell wall synthesis and subsequent lysis of a subpopulation of pneumococcal cells (Mutschler and Meinhart, 2011, 2013). The lysis of these cells would lead to the release of cellular components detrimental to the infected host such as pneumolysin. Interestingly, recent papers showed that a PezT/Zeta homolog, designated AvrRxo1 from the plant pathogen *Xanthomonas oryzae* pv. *oryzicola* functions as a type III-secreted virulence factor which is toxic in plants and is bacteriostatic when expressed in *E. coli* (Han et al., 2015; Triplett et al., 2016). An AvrRxo1 homolog from myxobacterium was also shown to trigger rapid cell death response in tobacco (Triplett et al., 2016). Intriguingly, although the AvrRxo1 toxin was found to be a nucleotide kinase, its target is not UNAG like PezT/Zeta but rather, the coenzyme nicotinamide adenine dinucleotide (NAD) and its biochemical precursor, nicotinic acid adenine dinucleotide (NAAD) leading to the formation of unusual 3′-phosphorylated products, 3′-NADP and 3′-nicotinic acid adenine dinucleotide phosphate (3′-NADDP) (Schuebel et al., 2016). A recent paper showed that 3′-NADP accumulates upon expression of AvrRxo1 in tobacco and rice leaves infected with AvrRxo1-expressing strains of *Xanthomonas oryzae*, thus indicating that the AvrRxo1 effector/toxin targets the coenzyme and redox carrier essential for central metabolic function of the host. However, the actual mechanism of 3′-NADP accumulation *in planta* is currently unknown as NAD and the conventional cofactor, 2′-NADP, are needed in 100s of essential reactions in the cell (Shidore et al., 2017). Hence, it could be possible that PezT/Zeta not only help in triggering the lysis of pneumococcal cells, the toxin itself may also be detrimental to the infected host cells. Indeed, it was recently shown that expression of the pneumococcal *pezT* toxin in the eukaryotic microalgae *Chlorella vulgaris* is lethal, leading to cellular damage and lysis (Ng et al., 2016). We await experimental results that would indicate if expression of PezT/Zeta in mammalian cells would be equally detrimental.

## Functional Overlaps Between Chromosomal and Plasmid-Encoded TA Systems

The second example is related to the role of TAs of *Salmonella enterica* serovar Typhimurium carrying the virulence plasmid pLST during bacterial infection (Lobato-Márquez et al., 2016b). One of the two TAs encoded by plasmid pLST is *vapBC*_ST_. This particular TA contributes to the successful colonization of recipient cells during infection, in conjunction with other type I and type II TAs encoded by the *Salmonella* chromosome (Lobato-Márquez et al., 2015). In addition to its role during infection, the plasmidic copy of *vapBC*_ST_ contributes to the maintenance of the plasmid (Lobato-Márquez et al., 2016b). Interestingly, the chromosomal copy of this particular TA seems to be inactive, so that the role in infection was taken up by the plasmid-encoded copy. Curiously enough, the VapC toxin of vapBC2_ST_ is active as a toxin, indicating that stabilization of pLST could be due to PSK (Lobato-Márquez et al., 2015).

An interesting example showing that location is compatible with different functions is provided by the first type II locus described, the *ccdAB*_F_ operon encoded by plasmid F (Jaffe et al., 1985). This operon was reported to contribute to plasmid maintenance by killing plasmid free-segregants; PSK was the result of Lon protease-mediated degradation of the CcdA antitoxin and the subsequent activation of the anti-topoisomerase activity of the toxin CcdB (Ogura and Hiraga, 1983). In addition to its role in plasmid maintenance, *ccdAB*_F_ was shown to contribute to bacterial persistence (Tripathi et al., 2012), a role that was also proposed for several chromosomal TA systems (Maisonneuve et al., 2011) and that has been questioned as reductionist recently (Ramisetty et al., 2016; Van Melderen and Wood, 2017). Furthermore, the *ccdAB*_ST_ system of plasmid pLST seems to participate in plasmid maintenance beyond PSK because, in spite of carrying a single point mutation that inactivates the anti-topoisomerase activity of CcdB_ST_, it contributes significantly to the stabilization of the virulence plasmid by a yet to be identified mechanism (Lobato-Márquez et al., 2016b). Functional interactions between co-existing *ccd* systems in plasmid and chromosome reported to be present in the pathogenic *E. coli* strain O157:H7 added further versatility to this system (Wilbaux et al., 2007). The chromosomally encoded *ccdAB* genes, like the plasmidic ones, has a toxin that target DNA gyrase and an antitoxin that is degraded by the Lon protease; however, both TAs seem to have evolved to achieve different functions: only the plasmidic antitoxin is able to neutralize the chromosomal toxin but not *vice versa*, and only the plasmidic TA is able to promote plasmid maintenance by PSK (Wilbaux et al., 2007). Nevertheless, a recent paper has shown that the chromosomal *ccdAB*_O157_ system from *E. coli* O157:H7 also function in the formation of persister cells much like its F-plasmid-encoded counterpart even though the CcdB_O157_ toxin displayed lower toxicity and has fivefold lower affinity for DNA gyrase compared to CcdB_F_ (Gupta et al., 2017).

Plasmid maintenance linked to the coordination of TAs with plasmid replication was initially reported by the Diaz-Orejas laboratory on the *kis-kid* TA encoded by plasmid R1 (Ruiz-Echevarría et al., 1995a,b), and further analyzed (Pimentel et al., 2005; López-Villarejo et al., 2012, 2015). Additional work revealed a further coordination of the *kis-kid* TA with cell cycle functions (Pimentel et al., 2014). On the whole the above work supports that, failures in plasmid R1 replication reduces the levels of the Kis antitoxin and increases the activity of the Kid toxin. This results in: (i) the rescue of plasmid replication mediated by Kid-dependent decrease in the expression levels of CopB, a secondary inhibitor of plasmid replication, and (ii) the decrease in the levels of key cell division proteins that allows the rescue of plasmid replication before cell division can occur.

Targeting by RNase toxins of mRNAs, tRNAs and rRNAs impact and remodel protein synthesis and are key to the stress response mediated by TAs (reviewed by Moll and Engelberg-Kulka, 2012; Cruz and Woychik, 2015). Targeting tRNA and remodeling of protein synthesis profile seem to be a general mechanism of stress response. Indeed, a recent publication (Chionh et al., 2016) reveals a mechanism related to response to oxidative stress and induction of persistence in *Mycobacterium bovis*. The mechanism implies modification of the tRNA anticodons for threonine or leucine. Due to these modifications, the tRNA will enable the efficient translation of particular proteins related to the oxidative stress response. These will lead, in turn, to: (i) remodeling the protein synthesis potential of the cell to respond to oxidative stress; (ii) preferential synthesis of a set of stress response proteins, and (iii) induction of persistence. Most interestingly these stress response mechanisms seem to be universal and are shared by prokaryotes and eukaryotes. Induction of persistence by TAs in response to stress also involves reduction of the protein synthesis potential and selective synthesis of proteins required to achieve survival to the stress-inducing agent (reviewed by Moll and Engelberg-Kulka, 2012).

## Concluding Remarks

TAs were proposed to be part of the accessory genome but as more and more details of their biological function are uncovered, the importance of these (apparently) expendable genetic entities to the lifestyle of their hosts are becoming clearer. As we have shown in this review, the biological functions of TAs do overlap irrespective of their location in their host genome – i.e., whether they are chromosomally encoded or plasmid-borne. Initially implicated in maintaining the stability of plasmids via PSK, TAs have since been shown to mediate the stability of genomic islands and even chromosome II of *V. cholerae* by PSK. Both plasmid- and chromosomally encoded TAs have also been implicated in persistence and virulence of several pathogens. It is thus clear that these hitherto “expendable” genetic loci have successfully integrated into their hosts’ cellular regulatory network, enabling their hosts to better adapt to their distinctive environmental niches.

## Author Contributions

RD-O, ME, and CCY designed the outline of the review. RD-O wrote the first draft, and all authors worked on it until the production of the final version.

## Conflict of Interest Statement

The authors declare that the research was conducted in the absence of any commercial or financial relationships that could be construed as a potential conflict of interest.
